# Building a Solid Case: Cigarette Smoking and Epigenomic Alterations

**DOI:** 10.1289/ehp.122-A194

**Published:** 2014-07-01

**Authors:** Wendee Nicole

**Affiliations:** Wendee Nicole was awarded the inaugural Mongabay Prize for Environmental Reporting in 2013. She writes for *Discover*, *Scientific American*, *National Wildlife*, and other magazines.

Smoking is a leading cause of premature death and disease worldwide,^1^ but figuring out just how it causes cancer and other diseases has proven more challenging. Several recent studies have shed light on one possible answer: Smoking modifies the epigenome, changing methylation patterns of genes, which in turn can alter gene expression. In this issue of *EHP*, a team of researchers at the National Institute of Environmental Health Sciences not only corroborates smoking-related associations previously reported for a number of CpG sites but also identifies new sites.^2^

CpG sites are cytosine and guanine nucleotides separated by a single phosphate that are found in gene promoter regions. Over the past few years, epigenome-wide association studies have suggested that smoking alters the methylation patterns of a number of CpG sites across the human genome.

**Figure:**
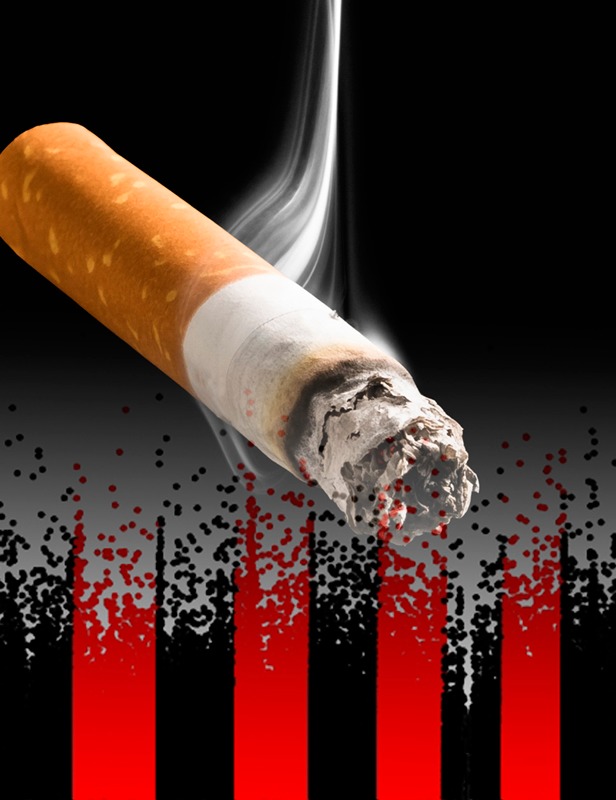
Epigenome-wide association studies are zeroing in on a number of CpG sites whose methylation patterns appear to be altered in response to smoking. © ArtBitz/Shutterstock

“When a cell divides, the daughter cell copies the methylation marks that the original cell had, and that can continue to influence whether a gene is transcribed even though the primary DNA sequence is not altered,” says Jack Taylor, head of the NIEHS Molecular and Genetic Epidemiology Section, who oversaw the study. If an environmental exposure can alter the epigenetic profile of the DNA, in turn influencing which genes are transcribed and affecting risk of disease, “that’s a big deal,” Taylor says.

In the current paper, the authors compared DNA methylation and history of cigarette smoking using data collected from the NIEHS Sister Study.^3^ This landmark project involves a cohort of more than 50,000 women whose sisters were previously diagnosed with breast cancer.

The study confirmed smoking-related associations for 10 previously identified CpG sites. Two CpG sites of particular interest are located on the *AHRR* and *CPOX* genes. *AHRR* is a tumor suppressor that also detoxifies polyaromatic hydrocarbons and regulates metabolism of dioxin, while the *CPOX* gene is involved in synthesis of heme (a component of hemoglobin). Smoking is known to increase heme synthesis, and the authors theorize that smoking might lead to increased expression of this gene and altered methylation.

“The Taylor article is a good addition to the body of evidence to support the previously identified markers, and identifies two new loci that could be of important biological interest,” says Natalie Shenker, a clinical researcher in the Epigenetics Unit at Imperial College London. “These two loci were also on our list of top hits,^4^ but did not reach the stringent cut-off for significance in our statistical analysis.”

The current study found that all 12 CpG sites identified showed a consistent trend of increasingly altered methylation from current smokers to past smokers to never-smokers, suggesting that methylation patterns may self-restore to some extent after a person quits. It also provides further evidence that DNA methylation patterns may serve as accurate long-term biomarkers for smoking.
